# Intelligence, Correspondence, &c.

**Published:** 1832-07-01

**Authors:** 


### INTELLIGENCE, CORRESPONDENCE, &c.

### NITRIC ACID IN TOOTH-ACHE.

*Hatton Garden, June 18th,* 1832.

DEAR SIR,

Your correspondent, Mr. Thomas, of Pembroke Dock, has, in your last number, claimed the discovery, of nitric acid as an efficacious remedy for tooth-ache, and informed us that he had used it in 1820, eight years before Mr. Myers mentioned it to me. I have no doubt of the truth of this statement, but must observe that several of my friends have assured me, they had known the acid applied to carious teeth many years ago; and therefore your correspondent's claim to the discovery is not better than my own. My object in publishing my paper was to recommend a trial of the remedy, as I had no doubt of its value, and to promote the interests of science and humanity. It appears rather singular that Mr. Thomas withheld his discovery for a period of eight years, and then published it after another had anticipated him. I am happy that he bears testimony in favour of its efficacy; and I take this opportunity of stating that repeated experience since the publication of my paper enables me to recommend it as an immediate palliative for the violent pain produced by caries of the teeth. I have never known it fail when applied to decayed teeth in the lower jaw, but it sometimes fails when disease is in the upper, from the difficulty of placing it in contact with the dental nerve.

I am, dear Sir,
Your's very truly,
M. RYAN.

---

We direct attention to a very able article in the Lancet for June 23d, by Mr. Fergus, relative to the late epidemic cholera in Vienna and other parts of Germany. Also to a communication in the same journal, from Mr. Murphy of Liverpool, on the outbreak of cholera in that great city.

Some very curious and instructive papers have also been communicated to our contemporaries by the Central Board, respecting Saline Injections in Cholera—all tending to shew that immense quantities of fluids may be safely thrown into the vascular system through a vein in the arm, while saline matters injected into the intestines or taken by the mouth, are worse than useless in Cholera. At present we will not venture a surmise as to the ultimate success of this practice. But we earnestly impress on our brethren and the public, the importance of guarding against *bowel-complaints,* to which there is now a strong tendency. CHOLERA is neither more nor less than EXCESSIVE DIARRHŒA.

